# Beneficial Effects of Anticoagulants on the Clinical Outcomes of COVID-19 Patients

**DOI:** 10.3390/antibiotics10111394

**Published:** 2021-11-13

**Authors:** Zubia Jamil, Azmat Ali Khan, Samreen Khalid, Muhammad Asghar, Khalid Muhammad, Yasir Waheed

**Affiliations:** 1 Department of Medicine, Foundation University Medical College, Foundation University Islamabad, Islamabad 44000, Pakistan; zubiajamil321@gmail.com (Z.J.); samreendoctor@gmail.com (S.K.); 2 Pharmaceutical Biotechnology Laboratory, Department of Pharmaceutical Chemistry, College of Pharmacy, King Saud University, Riyadh 11451, Saudi Arabia; azkhan@ksu.edu.sa; 3 Division of Infectious Diseases, Department of Medicine Solna, Karolinska Institutet, 17177 Stockholm, Sweden; asghar.muhammad@ki.se; 4 Department of Infectious Diseases, Karolinska University Hospital, 17164 Stockholm, Sweden; 5 Department of Biology, College of Sciences, United Arab Emirates University, Al Ain 15551, United Arab Emirates; k.muhammad@uaeu.ac.ae; 6 Multidisciplinary Lab, Foundation University Medical College, Foundation University Islamabad, Islamabad 44000, Pakistan

**Keywords:** COVID-19, SARS-CoV-2, heparin, enoxaparin, acute respiratory distress syndrome, pulmonary embolism

## Abstract

(1) Background: Severe coronavirus disease can be complicated by a hypercoagulable state in conjunction with sepsis, increasing the risk of venous thromboembolism. This study aimed to observe the effect of anticoagulants on 30-day high-dependency unit (HDU) outcomes of moderate to severe coronavirus disease 2019 (COVID-19) patients of a tertiary care hospital at Rawalpindi, Pakistan. (2) Methods: A retrospective propensity-based case–control study was carried out to examine COVID-19 patients admitted to the HDU. Patient groups who did and did not receive anticoagulants were labeled as “anticoagulant” and “non-anticoagulant”, respectively. Case–control matching (1:1) was performed via propensity scores (calculated by a regression model). Kaplan–Meier and logrank analyses were used to study survival probability. Single predictors of outcomes were determined by Cox regression analysis. (3) Results: The anticoagulant group had elevated D-dimers, advanced age, more comorbidities and a higher frequency of severe disease compared to the non-anticoagulant group (*p* < 0.05). Therefore, 47 cases and 47 matched controls were selected based on their propensity scores. The primary endpoint was outcome (survived vs. died). The 30-day in-HDU mortality was 25.5% for cases and 61.7% for controls (*p* = 0.0004). The median time from admission to death was 16 days for the case group and 7 days for the control group (*p* < 0.0001). The 30-day mortality was 19.1% for the enoxaparin group and 16.4% for the heparin group (*p* > 0.05). Enoxaparin (therapeutic and prophylactic doses) and heparin (prophylactic dose) were found to be independent factors affecting the outcomes of these patients (*p* < 0.001). (4) Conclusions: Anticoagulants play a beneficial role in reducing mortality among COVID-19 patients. Both anticoagulant formulations, enoxaparin (therapeutic and prophylactic doses) and heparin (prophylactic dose), were associated with improving survival among these patients.

## 1. Introduction

Coronavirus disease 2019 (COVID-19), caused by severe acute respiratory syndrome coronavirus 2 (SARS-CoV-2), has become a major public health burden worldwide. It has resulted in a frightening increase in mortality around the world. As of 1 November 2021, over 247 million confirmed cases of COVID-19 have been reported worldwide, including 5.02 million deaths [1]. Currently, Pakistan has had 1.27 million confirmed cases of COVID-19, with 28,466 deaths so far [2].

New research in this field proves that severe coronavirus disease can be further complicated by a prothrombotic state, resulting in an increased risk of venous thromboembolism of up to 30% among patients in intensive care [3]. SARS-CoV-2 induces an inflammatory reaction from a cytokine storm, which activates the coagulation cascade, producing increased levels of thrombin and fibrin degradation products. This results in disseminated intravascular coagulation with a thrombotic tendency. The deposition of fibrin along with an injured lung endothelium predisposes a patient to microvascular thrombosis, contributing to hypoxemia and causing acute respiratory distress syndrome, which results in prolonged mechanical ventilation, a guarded prognosis and death [4].

The laboratory parameters, which include D-dimers, prothrombin time, platelet count and inflammatory biomarkers (including interleukin-6, C-reactive protein, ESR, procalcitoninin and ferritin), not only serve as markers of disease severity but can also guide decision making for early admission to the intensive care unit (ICU), intubation and ventilator therapy as well as the need for anticoagulation, in addition to assisting in determining the outcomes of COVID-19 patients [5]. Recent studies have indicated that anticoagulant therapy can improve survival in COVID-19 patients with high D-dimer levels and suspected hypercoagulability [3].

The International Society on Thrombosis and Hemostasis and the American Society of Hematology recommend that all critically ill COVID-19 patients should be treated with prophylactic dose anticoagulants to prevent microcirculation thromboembolism [6]. The American College of Cardiology has also proposed anticoagulant treatment in these patients [7]. However, further studies are needed to evaluate the beneficial effect of the use of anticoagulants in critically ill COVID-19 patients, as well as the type of anticoagulant and method of administration in different populations worldwide. 

Unfractionated heparin (UFH) or low-molecular-weight heparin (LMWH) are used in the management of severe COVID-19 patients. Besides having anticoagulant effects, these agents have anti-inflammatory and cytoprotective effects [8]. Direct oral anticoagulants are also being used to treat COVID-19 hypercoagulability, but the outcomes have been unaffected [9]. Although both UFH and LMWH can be used in these settings, due to the unwarranted need to monitor activated partial thromboplastin time (aPTT) and the lower potential to induce bleeding and thrombocytopenia in comparison to UFH, LMWH may be preferred [10,11].

The aim of this study is to analyze the effects of parenteral anticoagulants (unfractionated heparin and enoxaparin) in therapeutic or prophylactic doses as well as oral anticoagulants (rivaroxaban) among moderate to severe COVID-19 patients with coagulopathy, admitted to the HDU of our tertiary care hospital at Rawalpindi, Pakistan.

## 2. Results

**Figure 1:**
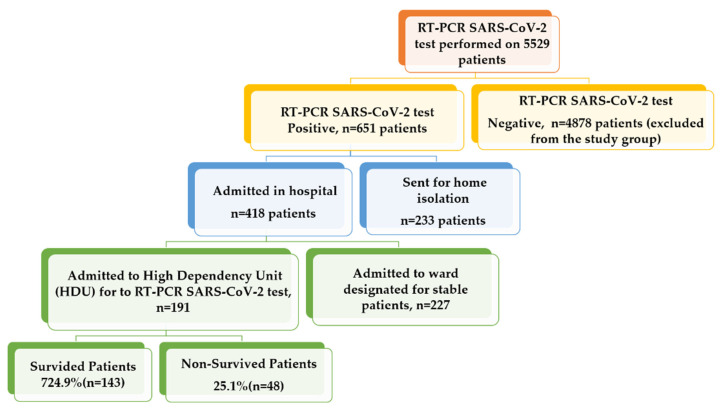
The flowchart showing the distribution of COVID-19 patients among different sections in sequential order.

A total of 191 patients admitted to the HDU with moderate to severe COVID-19 disease were included in this study. A flowchart describing the distribution of patients is shown in Figure 1.

This tertiary care hospital is meant to provide medical facilities to families of lower-rank soldiers coming from suburbs of the capital city and belonging to lower socio-economic backgrounds. Gender-wise, an examination revealed that 75.9% (*n* = 145) of the patients were female, which was higher than the 24.1% of patients who were male (*n* = 46). The mean age was 54.25 + 15.85 (14–88) years. We found that patients with comorbid conditions became seriously ill due to COVID-19 compared to those who did not have comorbid conditions. About 71.7% (*n* = 137) of the patients who needed oxygen inhalation and admission to the HDU had comorbid conditions and 28.3% (*n* = 54) did not have any comorbid conditions. The most common comorbid condition resulting in admission to the intensive care unit was diabetes mellitus, found in 57.6% of the patients (*n* = 110). Other conditions were hypertension, in 49.7% (*n* = 95), chronic kidney disease, in 14.1% (*n* = 27), ischemic heart disease, in 8.9% (*n* = 17), chronic obstructive pulmonary disease, in 7.3% (*n* = 14), cerebrovascular accidents, in 6.3% (*n* = 12), congestive cardiac failure, in 6.3% (*n* = 12), chronic liver disease due to hepatitis C infection, in 4.2% (*n* = 8) and solid tumors in addition to the use of immunosuppressive drugs, in 3.1% (*n* = 6).

Diabetes complicated by diabetic ketoacidosis was present in 3.7% (*n* = 7) of the patients while 1.5% (*n* = 3) of diabetic patients had mucormycosis with fungal meningitis. A trivial number of patients had hypothyroidism, 1% (*n* = 2), systemic lupus erythematosus (SLE) with lupus nephritis, 1% (*n* = 2), necrotizing pancreatitis 0.5% (*n* = 1), neuro-brucellosis, 0.5% (*n* = 1) and epilepsy, 0.5% (*n* = 1).

### 2.1. Patients’ Characteristics

The main purpose of this study was to evaluate the role of anticoagulants in the survival of COVID-19 patients. The anticoagulants that are studied in this manuscript are parenteral anticoagulants (unfractionated heparin and enoxaparin) in therapeutic and prophylactic doses as well as oral anticoagulants (rivaroxaban); therefore, in the rest of the manuscript the term “anticoagulants” will refer to these formulations of anticoagulants. First, the study cohort was broadly classified into two groups: the anticoagulant group, who received anticoagulants (68.1%, *n* = 130), and the non-anticoagulant group, who did not receive anticoagulants due to contraindications (31.9%, *n* = 61).

Patients with thrombocytopenia (a platelet count < 50,000/µL) were not given anticoagulants. Important causes of thrombocytopenia were portal hypertension due to chronic liver disease (*n* = 8), immune-mediated thrombocytopenic purpura (*n* = 7), aplastic anemia (*n* = 4), myelodysplastic syndrome (*n* = 3), post chemotherapy (*n* = 3) and chronic lymphocytic leukemia (*n* = 1). Anticoagulants were also not given to patients with hemorrhagic stroke (*n* = 11), gastrointestinal bleeding (*n* = 10), post major surgical procedures (*n* = 5), intracranial tumors (*n* = 3), post ophthalmic surgery (*n* = 2), hemolysis, elevated liver enzymes and low platelets (HELLP) syndrome (*n* = 1), fulminating hepatic failure (*n* = 1) and the presence of cerebral arterial aneurysm (*n* = 1).

### 2.2. Propensity-Score-Based Case–Control Matching

The examination of the biochemical characteristics of the two groups (anticoagulant vs. non-anticoagulant group) revealed that many parameters were significantly different between the groups. The age of patients, presence of comorbid conditions (diabetes mellitus and hypertension), disease severity, platelet count and D-dimer as well as troponin T levels were significantly different among these groups (Table 1). Case–control matching was undertaken using a propensity-score-based approach in a ratio of 1:1 to address these confounders. A total of 47 cases and 47 matched controls were selected. 

Case–control matching revealed no significant differences in comorbid conditions, disease severity or biochemical characteristics between cases and controls (all *p* > 0.05, Table 1). Comparing the survival rates of the two groups, the case group exhibited a higher survival rate of 74.4%, while the control group showed only a 38.3% survival rate (RR = 12.32, 95% CI = 16.12–52.29 and *p* < 0.0001). The comparison of various parameters among the study cohort is shown in Table 1.

**Figure 2:**
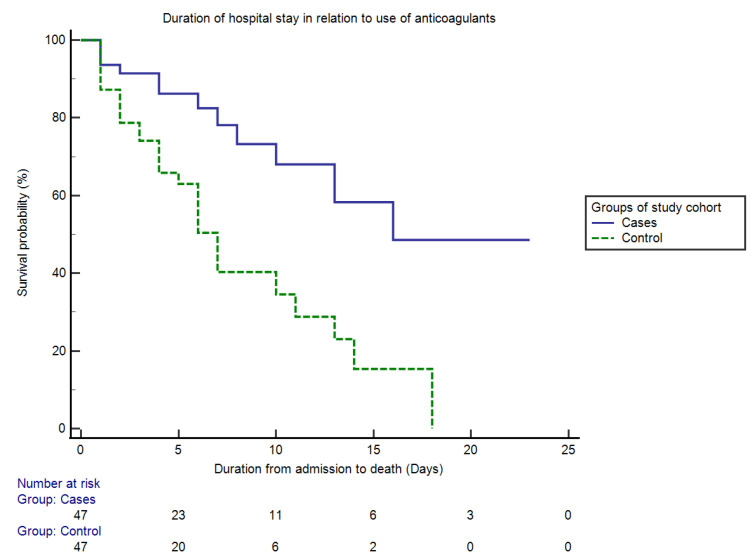
Kaplan–Meier curve showing the overall survival probability from hospital admission to death among two groups of the study cohort (cases vs. controls) in relation to the use of anticoagulants. The Kaplan–Meier analysis revealed that the 30-day HDU mortality for the case group was only 25.5%, compared with 61.7% for the control group (*p* < 0.05). An average time spent in the hospital was 16 days for the case group and 7 days for the control group (*p* < 0.05).

The primary endpoint of this study was outcome (survived vs. died). Among the 191 patients admitted to the HDU, 74.9% (*n* = 143) of them survived and 25.1% (*n* = 48) did not. The mean duration of hospital admission till an outcome was 6.85 + 4.99 days. The 30-day in-HDU mortality was only 25.5% (12 out of 47 patients) for the case group vs. 61.7% (29 out of 47 patients) for the control group (RR = 12.39, 95% CI = 16.22–52.38 and *p* = 0.0004). In the case group, the median hospital stay (from hospital admission to death) was 16 days (SE = 1.67, HR = 0.31 and 95% CI = 12.32–18.89); in the control group, it was 7 days (SE = 1.02, HR = 3.20, 95% CI = 6.18–10.16). The difference in duration between the two groups was statistically significant (logrank × 2 = 12.49, *p* < 0.0001). A Kaplan–Meier curve showing the overall survival probability in relation to the use of anticoagulants is shown in Figure 2.

**Figure 3:**
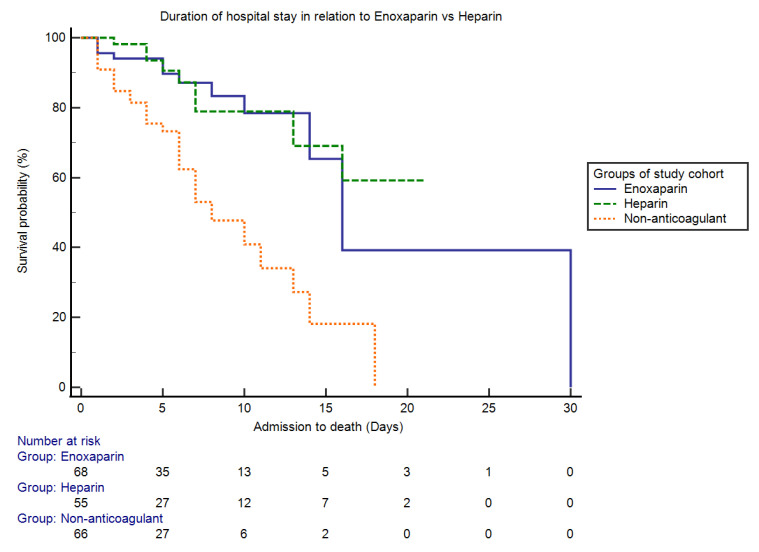
Kaplan–Meier curve showing the overall survival probability from hospital admission to death among the study cohort in relation to two groups of anticoagulants (enoxaparin vs. heparin). The Kaplan–Meier analysis showed that the 30-day in-HDU mortality was 19.1% for the enoxaparin group vs. only 16.4% for the heparin group (*p* > 0.05). The median time of hospital stay was 19 days for the enoxaparin group and 16 days for the heparin group (*p* < 0.05).

The anticoagulant group was divided into five subgroups, which were: Group I, enoxaparin in a therapeutic dose, 23% (*n* = 44); Group II, enoxaparin in a prophylactic dose, 13.1% (*n* = 25); Group III, heparin in a therapeutic dose, 9.9% (*n* = 19); Group IV, heparin in a prophylactic dose, 19.4% (*n* = 37); and Group V, oral anticoagulants, 2.6% (*n* = 5). We also studied the role of enoxaparin vs. heparin in determining the survival probability of COVID-19 patients during an HDU stay using Kaplan–Meier analysis. The 30-day in-HDU mortality was 19.1% (13 out of 68 patients) for the enoxaparin group vs. 16.4% (9 out of 55 patients) for the heparin group (RR = 0.15, 95% CI = −11.42–15.93 and *p* = 0.69). The median time of hospital stay (admission to death) was 19 days for the enoxaparin group (SE = 2.72, HR = 0.84 and 95% CI = 13.67–24.36) and 16 days for the heparin group (SE = 1.30, HR = 1.18 and 95% CI = 13.89–18.99) (logrank × 2 = 12.49, *p* < 0.0001). A Kaplan–Meier curve showing the overall survival probability from hospital admission to death among the study cohort in relation to two groups of anticoagulants, enoxaparin vs. heparin, is shown in Figure 3.

At the end, multivariate regression analysis was used to determine the factors affecting the outcomes of moderate to severe COVID-19 patients (*n* = 191). First, the factors were analyzed by linear regression. Only statistically significant factors were then analyzed by Cox regression analysis. A low platelet count, high troponin T levels, increasing age and the presence of multiple comorbid conditions were found to be predictors of survival in these patients. Anticoagulant formulations enoxaparin (therapeutic and prophylactic doses) and heparin (prophylactic dose) were independent factors associated with survival among these patients (*p* < 0.001). Patients with oral anticoagulation did not experience any advantage. Different factors in predicting the outcomes of COVID-19 patients according to Cox proportional hazards regression analysis are shown in Table 2.

## 3. Discussion

The COVID-19 pandemic has caused a major global health burden and increased mortality. This infectious disease has a wild range of clinical symptoms, starting from mild respiratory illness with fever, cough, myalgia, headache, anosmia and diarrhea to severe pneumonia with acute respiratory distress syndrome, cardiac arrhythmias, septic shock, hypercoagulability, disseminated intra-vascular coagulation with multi-organ failure and even death [12]. These clinical features help in identifying the patients suffering from SARS-CoV-2 infection even before the RT-PCR for COVID-19 comes back as positive; therefore, they help in managing these patients in a timely manner [13]. 

Thrombotic complications are observed in COVID-19 in two phenotypic forms: the diffuse micro-thrombotic type and the classical thromboembolic type [10]. Thrombosis in the microvasculature, along with inflammation (thus termed thromboembolism), is associated with venous and arterial thromboembolism, stroke, respiratory failure and even death [14,15]. There have been attempts to minimize the incidence of COVID-19 using strategies similar to this study, where ophthalmic solutions are being used as antiviral agents [16]. Early anticoagulation decreases the incidence of coagulopathy and mortality by reducing the chance of microthrombus formation and the risk of organ failure [17]. Heparin in both unfractionated and low-molecular-weight forms has been beneficial in controlling inflammation and thrombosis in this disease [18]. The current guidelines recommend a prophylactic dose of LMWH to be given to all COVID-19 pneumonia patients requiring hospitalization, in the absence of any contraindications [6,19]. Here, we studied the beneficial effects of parenteral anticoagulants (unfractionated heparin and enoxaparin) in therapeutic and prophylactic doses in addition to oral anticoagulants (rivaroxaban) in a total of 191 patients with moderate and severe COVID-19 disease who were admitted to the high-dependency unit of our tertiary care hospital at Rawalpindi, Pakistan.

In our study group, most of the patients with comorbidities developed severe illness and a guarded prognosis as compared to patients without comorbidities (*p* < 0.05), associated with higher D-dimer levels (*p* < 0.05), advanced age, platelet counts and high troponin levels in severe COVID-19 patients receiving anticoagulants as compared to the non-anticoagulant group (*p* < 0.05). A few patients presented with diabetic complications, such as diabetic ketoacidosis, mucormycosis with fungal meningitis and necrotizing pancreatitis. Our findings are in accordance with the previous investigation of critically ill COVID-19 patients by Zhou F et al. [20] and Tang N et al. [21], where the mortality rate was reported to be around 30% due to old age and multiple comorbidities alongside inflammatory biomarkers, including elevated D-dimers on admission, increased prothrombin time and high IL-6 as well as troponin T levels. A case study by Chan KH et al. [22] showed that diabetic patients are at an increased risk of developing complications associated with COVID-19, contributing to increased mortality. In a study by Berger JS et al. [23] on nearly two thousand COVID-19 hospitalized patients, around 76% had elevated D-dimers at presentation. Rates of adverse events increased with the magnitude of D-dimer elevation [15,24,25]. These were potential risk factors for severe infection and could help physicians identify patients with a poor prognosis at an early stage [20].

Patients with absolute or relative contraindications to anticoagulants were placed in the non-anticoagulant group (31.9%, *n* = 61). The main cause of contraindication in our study cohort was chronic liver disease due to hepatitis C infection (*n* = 8), resulting in either thrombocytopenia due to portal hypertension or coagulopathy due to hepatic insufficiency. This fact is also evident by a study conducted by Jacob A et al. [26], which showed an enhanced risk of bleeding in patients with chronic liver disease and coagulopathy.

Among the 191 patients admitted to the HDU, 74.9% survived while 25.1% died. This mortality rate was similar to a case study performed by Klok F A. et al. [27], who found that the mortality rate was nearly 20% in 180 critically ill patients. However, the mortality rate was higher (around 60%) in a study by Xu J et al. [28]. Further case–control matching in a ratio of 1:1 (47:47) was performed via propensity scores to address confounders such as biochemical parameters, disease severity and the presence of comorbid conditions. Our analysis showed that the case group receiving anticoagulants had an improved survival rate (74.4% vs. 38.3%, *p* < 0.0001). Moreover, the overall survival probability significantly increased in the case group (median hospital stay was 16 days vs. the 7 days of the control group) (Figure 2). These findings are consistent with a series of studies that claim anticoagulation to be of substantial benefit in improving clinical outcomes in severe COVID-19 patients [19,29,30].

We analyzed the efficacy of enoxaparin versus heparin, and found that among the subgroups of anticoagulants enoxaparin, in therapeutic and prophylactic doses, is an independent predictor of survival, while only a prophylactic dose of unfractionated heparin is significant in improving survival (Table 2). A longer in-hospital survival of 19 days was observed in patients who received enoxaparin compared to those who received heparin, with a median survival of 16 days (Figure 3). A few case studies showed that the use of a prophylactic dose of enoxaparin resulted in a significant reduction in mortality [6,10,11]. These beneficial effects of LMWH are not only due to its anticoagulant effects, but also its anti-inflammatory properties, which help to decrease elevated cytokines and pro-inflammatory markers in severe cases of COVID-19 [31]. However, a recently published study by Canoglu K et al. [32] showed that even a therapeutic dose of enoxaparin also decreases mortality in critically ill COVID-19 patients. It has been recommended that for COVID-19 patients with high D-dimers and imminent respiratory failure, intermediate or therapeutic dosing of LMWH should be considered, according to the bleeding risk [30]. Additionally, in COVID-19 patients with sepsis-induced coagulopathy, therapeutic dosing avoids progression to overt disseminated intravascular coagulation (DIC), and the use of enoxaparin is preferred to unfractionated heparin [5].

The Scientific and Standardization Committee suggests weight-based therapeutic doses of heparin are more effective than fixed dosing for thromboprophylaxis in high-risk subsets of patients hospitalized with COVID-19 [33]. Additionally, heparin appears to be the safest anticoagulant due to a low risk of interaction with current COVID-19 therapies [34]. However, oral anticoagulants did not improve the survival period of patients as compared to the control group in our study. This is supported by Schiavone M et al. [35], where oral anticoagulants appeared to be ineffective in reducing mortality rates in COVID-19 patients.

The main limitation of this study was that the anticoagulant group had significantly different characteristics from the non-anticoagulant group in terms of disease severity, comorbid conditions and other biochemical parameters. Although this confounder was addressed by propensity-based case–control matching, after matching the sample size became trivial (47:47). Despite the major limitations, such as the retrospective, single-center study design and limited sample size, this is one of the first studies from a Third World country to determine the efficacy of using anticoagulants in moderately to severely affected COVID-19 patients in real-world hospital settings.

## 4. Materials and Methods

The current study was carried out from December 2020 to August 2021 at the COVID-19 HDU at a tertiary care hospital, Rawalpindi, Pakistan.

### 4.1. Study Cohort and Inclusion Criteria

In the given period, a total of 5529 suspected COVID-19 cases were examined for SARS-CoV-2 infection. All patients that had confirmed SARS-CoV-2 infection by real-time reverse transcription polymerase chain reaction (RT-PCR) and required admission to the HDU were included. Patients were transferred from the emergency, general, medical and COVID-19 stable wards to the HDU. The criteria for HDU admission were as follows:

- Patients with moderate disease (SpO_2_ > 90%, but <94%) whose chest X-ray shows infiltrates in <50% of total lung fields or whose high-resolution chest CT shows peripheral ground glass opacities.
- Patients with severe disease (SpO_2_ < 90% + stable vital signs, respiratory rate > 30/min and chest X-ray showing infiltrates >50% of total lungs) or high-resolution chest CT showing extensive peripheral ground glass opacities.

Severe COVID-19 disease was further categorized into five phases [2]:

1. Lung involvement alone, evident by chest X-ray or HRCT;
2. Cytokine release syndrome (CRS), which is a systemic inflammatory response that can be triggered by a variety of factors, such as infections and certain drugs. CRS was diagnosed by a documented rise in inflammatory markers (CRP > 70 mg/L, LDH > 300 U/L, ferritin > 700 ng/mL and D-dimers > 1000 ng/mL (or > 1 mcg/mL)), with a rising tendency of these markers in the last 24 h;
3. Evidence of myocarditis (electrocardiogram changes, positive troponin T, elevated proBNP or echo findings);
4. Sepsis with or without septic shock;
5. Combination of above phases.

The study group in which anticoagulants were given were labeled as the “anticoagulant group”. The anticoagulants that were studied in this manuscript are parenteral anticoagulants (unfractionated heparin and enoxaparin) in therapeutic and prophylactic doses and oral anticoagulants (rivaroxaban); so, in the whole manuscript, the term “anticoagulants” refers to these formulations of anticoagulants.

### 4.2. Exclusion Criteria

The following patients were excluded from the study group:

- Those COVID-19 patients who had mild symptoms and were advised to isolate at home;
- Those patients who were admitted to the stable COVID-19 ward.

The anticoagulant group received the mentioned formulations of anticoagulants with the following doses [2]:

- Group I: enoxaparin in a therapeutic dose (1 mg/kg × 12 hourly) subcutaneously;
- Group II: enoxaparin in a prophylactic dose (40 mg × once a day) subcutaneously;
- Group III: heparin in a therapeutic dose (80 units/kg loading dose, then 18 units/kg/hour);
- Group IV: heparin in a prophylactic dose (5000 units × 12 hourly) subcutaneously;
- Group V: oral anticoagulants (rivaroxaban, 15–20 mg, once a day).

The following criteria were used for administrating anticoagulants [2]:

### 4.3. Therapeutic Dose of Enoxaparin or Heparin (Group I and Group III)

- D-dimer levels more than 3 times the upper limit;
- Documented presence of thromboembolic disease (Doppler ultrasound for deep vein thrombosis or CT scan findings for pulmonary embolism);
- Strong clinical suspicion of thromboembolic disease, even without investigation.

### 4.4. Prophylactic Dose of Enoxaparin or Heparin (Group II and Group IV)

Patients with moderate or severe disease who did not meet the above criteria received prophylactic anticoagulant therapy.

### 4.5. Oral Anticoagulants (Group V)

Patients who were already on oral anticoagulants (rivaroxaban) due to any indication, such as atrial fibrillation, mitral stenosis, reduced ejection fraction, valve replacement, etc., proceeded with the same dose of oral anticoagulants, provided they did not fall into the category of therapeutic treatment with parenteral anticoagulants as mentioned in Section 4.3.

The choice between heparin or enoxaparin was made depending on creatinine clearance (CrCl). Heparin was used in patients with CrCl < 30 mL/min and enoxaparin was used with CrCl > 30 mL/min. All the patients in which anticoagulants were not used due to absolute or relative contraindications were taken as the “non-anticoagulant group”.

Patients with thrombocytopenia (platelet count < 50,000/µL) due to any cause were not given anticoagulants. Anticoagulants were also not given to patients with hemorrhagic stroke, gastrointestinal bleeding, post major surgical procedures, intracranial tumors, post ophthalmic surgery, hemolysis, elevated liver enzymes and low platelets (HELLP) syndrome, fulminating hepatic failure and the presence of cerebral arterial aneurysm.

### 4.6. Study Method

Approval was received from the ethics board before conducting the study. About 191 patients were admitted to the HDU during this period. SARS-CoV-2 infection was confirmed via RT-PCR by taking a sample through a nasopharyngeal swab. Our hospital has a Medix software system for keeping patient records, clinical data and details of the patient’s management. At the time of admission, history and vital signs, including blood pressure, pulse, temperature and oxygen saturation, were noted in the system. All the laboratory data can be retrieved from this system by the specific admission record number given to the patient at the time of admission. A Doppler ultrasound of leg veins and a chest high-resolution CT or a pulmonary angiography CT were also done in selected patients. Written informed consent was obtained by hospital authorities from every patient or the attendants (first-degree blood relatives) of critically ill patients.

### 4.7. Propensity-Based Case–Control Matching

Several biochemical and clinical parameters among patients who required anticoagulants, as opposed to the non-anticoagulant group, were analyzed by propensity-based case–control matching. First, all the variables were checked by linear regression analysis. Propensity scores were calculated by regression analysis for those variables that affected the outcomes of these patients. Based on the calculated propensity scores of regression analysis, case–control matching was done (1:1). In this way, case–control matching was not only conducted by selecting variables, but also by all variables that could affect the outcomes of these patients.

### 4.8. Statistical Analysis

MedCalc Statistical Software 19.6.4 (MedCalc software, Ostend, Belgium) and SPSS, version 26, were used for statistical analysis. Quantitative variables were expressed as means, standard deviations and ranges, while qualitative variables were expressed as percentages. Patients were divided into two groups according to the use of anticoagulants: anticoagulant vs. non-anticoagulant groups. The controls were matched with cases of the study cohort by propensity-based matching in a ratio of 1:1. Quantitative variables of the two groups were compared by Student’s t-tests, and qualitative variables were compared by chi-square tests. For 30-day survival probability analysis, Kaplan–Meier and logrank analyses were used in relation to different groups of the study group. At the end, single predictors of survival were determined by Cox regression analysis.

## 5. Conclusions

In conclusion, our study demonstrated that anticoagulants had a significant impact on the mortality rate of severe to moderate COVID-19 patients and are imperative for managing these patients due to a high frequency of micro-, venous and arterial thromboembolism. Depending on D-dimers and the risk of pulmonary embolism, both anticoagulant formulations, enoxaparin (therapeutic and prophylactic doses) and heparin (prophylactic dose), were associated with improving survival among these patients. However, patients’ survival rates have not been observed to be improved by oral anticoagulants.

## Figures and Tables

**Table 1:** Comparison of biochemical parameters, comorbid conditions, disease severity and survival of COVID-19 patients. Coagulant vs. non-anticoagulant group and cases vs. control in relation to *p*-value.

	Before Matching			After Matching		
Variables	Anticoagulant Group (*n* = 130)	Non-Anticoagulant Group (*n* = 61)	*p*-Value	Cases (*n* = 47)	Controls (*n* = 47)	*p*-Value
Biochemical parameters						
Age	47.40 + 13.54	54.37 + 11.89	0.00	53.87 + 12.57	52.20 + 15.69	0.56
Platelets × 10^3^ cells/L	246.42 + 95.05	125.46 + 82.89	0.01	201.57 + 25.15	187.34 + 32.59	0.21
D-dimers (ng/mL)	367.97 + 91.17	122.31 + 32.21	0.01	245.92 + 95.26	221.3 + 82.24	0.38
Trop T (ng/mL)	0.33 + 0.24	0.08 + 0.64	0.02	0.20 + 0.14	0.18 + 0.34	0.22
Comorbidities			0.00			
Yes	82.3% (107/130)	57.4% (35/61)		63.8% (30/47)	65.9% (31/47)	0.62
No	17.7% (23/130)	42.6% (26/61)		36.2% (17/47)	34.1% (16/47)	
Diabetes mellitus			0.00			
Yes	66.2% (86/130)	47.5% (29/61)		46.8% (22/47)	48.9% (23/47)	0.16
No	33.8% (44/130)	52.5% (32/61)		53.2% (25/47)	51.1% (24/47)	
Hypertension			0.00			
Yes	60% (78/130)	39.3% (24/61)		46.8% (22/47)	48.9% (23/47)	0.58
No	40% (52/130)	60.7% (37/61)		53.2% (25/47)	51.1% (24/47)	
Disease severity			0.05			
Moderate disease	30% (39/130)	42.6% (26/61)		31.9% (15/47)	31.9% (15/47)	1.00
Severe disease	70% (91/130)	57.4% (35/61)		68.1% (32/47)	68.1% (32/47)	
Survival analysis			0.00			
Survived	84.6% (110/130)	54.1% (33/61)		74.4% (35/47)	38.3% (18/47)	<0.0001
Died	15.4% (20/130)	45.9% (28/61)		25.5% (12/47)	61.7% (29/47)	

Before propensity score matching the anticoagulant and non-anticoagulant groups were significantly different in relation to different parameters (biochemical parameters, presence of comorbidities, diabetes mellitus, hypertension and disease severity), which was adjusted by matching. Among cases only 25.5% of the patients died, compared to the control group in which 61.7% of the patients died.

**Table 2:** Table showing factors affecting the outcomes of moderate to severe COVID-19 patients according to Cox proportional hazards regression analysis.

Variables	OR (95% CI)	*p*-Value
Age	1.03 (1.01–1.05)	0.00
Platelet count	0.81 (0.74–0.89)	0.00
Troponin T levels	1.09 (1.03–2.01)	0.01
Comorbid conditions	0.32 (0.12–0.83)	0.02
Enoxaparin (therapeutic dose)	0.26 (0.11–0.65)	0.00
Enoxaparin (prophylactic dose)	0.19 (0.06–0.64)	0.00
Heparin (therapeutic dose)	0.60 (0.20–1.74)	0.34
Heparin (prophylactic dose)	0.05 (0.01–0.26)	0.00
Oral anticoagulants	0.26 (0.05–1.22)	0.09
Non-anticoagulant group	0.27 (0.06–1.17)	0.08

Footnotes: odd ratio (OR), confidence interval (CI), age, platelet count, troponin T levels, the presence of comorbid conditions, enoxaparin in both therapeutic or prophylactic doses and heparin in a prophylactic dose affect the outcomes of COVID-19 patients.

## Data Availability

Data is available on request to first author.
